# Supplementary material for: Transcriptome Analysis Reveals Vimentin-Induced Disruption of Cell–Cell Associations Augments Breast Cancer Cell Migration
Source: Cells. 2022 Dec 13;11(24):4035. doi: 10.3390/cells11244035 (PMC9776984; doi:10.3390/cells11244035)
Supplement: Supplementary file 1 [file cells-11-04035-s001.zip › DEGs and enrichment analysis.zip!/DEGs and enrichment analysis/WT_VIMvsVector KEGG/DOWN/WT_VIMvsVector_DOWN.pathway_map.zip!/WT_VIMvsVector_DOWN.pathway_map/src/hsa05212.html]

hsa05212


- hsa:7042

- Down regulated genes

ENSG00000092969(-2.32734226266555)

Close
